# Primary pancreatic lymphoma: Report of three cases with review of literature

**DOI:** 10.4103/0971-5851.56331

**Published:** 2009

**Authors:** Altaf Gauhar Haji, Shekhar Sharma, K. Abdul Majeed, D. K. Vijaykumar, K. Pavithran, M. Dinesh

**Affiliations:** *Department of Surgical Oncology, Amrita Institute of Medical Sciences and Research Center, Ernakulam, Kerala, India*; 1 *Department of Medical Oncology, Amrita Institute of Medical Sciences and Research Center, Ernakulam, Kerala, India*; 2 *Department of Radiation Oncology, Amrita Institute of Medical Sciences and Research Center, Ernakulam, Kerala, India*

**Keywords:** *Pancreatic lymphoma*, *pancreatic adenocarcinoma*, *neoplasm*

## Abstract

**Background:**

Primary pancreatic lymphoma (PPL) is an extremely rare neoplasm, which may be confused with pancreatic adenocarcinoma. So far only about 150 cases of PPL have been reported.

**Materials and Methods:**

We present our experience of 3 cases of PPL over a 4-year period.

**Results:**

All the patients presented with vague abdominal pain of duration ranging from 1½ months to 3 months. Two patients had diagnosis confirmed histologically by CT-guided core biopsy or Fine needle aspiration procedure. We were able to avoid unnecessary laparotomy in 2 patients using preoperative guided Fine needle aspiration Cytology, although the third patient did undergo a Whipple′s procedure as the diagnosis of PPL was not considered during the initial workup.

**Conclusions:**

There is no significant difference noted with regard to patient′s age or duration of symptoms between patients with either pancreatic adenocarcinoma or PPL. The differential diagnosis of PPL includes pancreatic adenocarcinoma and secondary involvement of pancreas from extra-nodal lymphoma. Combination of two things is suggestive of Pancreatic lymphoma: (1) Bulky localized tumor in pancreatic head (2) Absence of significant dilatation of main pancreatic duct strengthens a diagnosis of pancreatic lymphoma over adenocarcinoma. Majority of patients can be managed with chemotherapy with much better prognosis compared to patients with pancreatic adenocarcinoma. Larger series of patients are needed to evaluate whether chemotherapy, eventually followed by involved-field radiation therapy, is the treatment of choice for PPL.

## INTRODUCTION

Gastrointestinal (GI) tract is the most common site of extra-nodal non-Hodgkin′s lymphomas (NHLs), accounting for 15% to 20% of all NHL cases.[1] Although secondary involvement of pancreas is seen often in cases of GI lymphoma, primary pancreatic lymphoma (PPL) is an extremely rare disease that can mimic pancreatic carcinoma.[1,2] Fewer than 2% of extra-nodal malignant lymphomas and 0.5% of all pancreatic masses constitute PPL.[3,4] Significant, however, is the fact that PPLs are potentially treatable.[5]

To date, approximately 150 cases of PPL have been reported in the English-language medical literature.[6] There is no published data on PPL in the Indian population except for a single case report.[7]

## MATERIALS AND METHODS

We present our experience of treating primary pancreatic lymphoma in patients at our hospital. Medical records of patients identified during a retrospective review of prospectively maintained database in the cancer institute of our hospital were retrieved after obtaining informed consent from the concerned patients. A pathologist reviewed slides and blocks to reconfirm the diagnosis. Literature was accessed on the Internet through PubMed search using the string 'primary pancreatic lymphoma.'

## RESULTS

**Figure 1:**
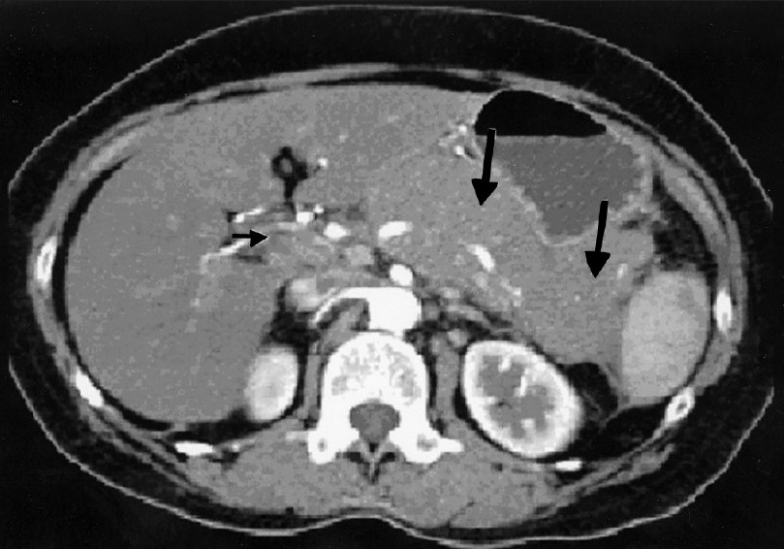
CT scan showing diffuse homogenous enlargement of pancreatic parenchyma (large arrow) with regional nodal disease (small arrow)

Over a period of 4 years, we have diagnosed and treated 3 cases of primary pancreatic lymphoma [Table 1]. All the 3 patients presented with vague abdominal pain of duration ranging from 1½ months to 3 months. Other symptoms reported include low-grade fever in 2 patients, weight loss in 1, loss of appetite in 1 and melena in 1 patient. CT scan showed hypodense mass in body of pancreas (head and tail also in 1 case) encasing the vessels, peri-pancreatic lymph node enlargement with no bile or pancreatic duct dilatation [Figure 1] in all cases. These features are not very typical of a pancreatic malignancy. Two patients had diagnosis confirmed histologically by CT-guided core biopsy or FNA procedure. One patient underwent Whipple′s procedure before the diagnosis was made.

**Figure 2:**
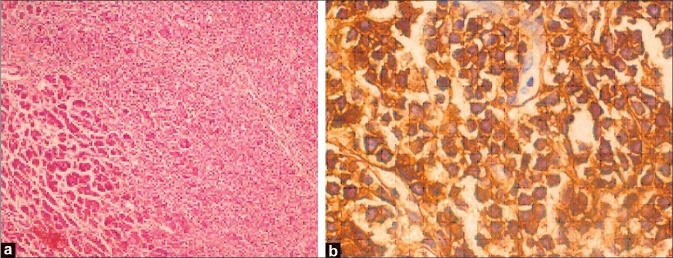
Photomicrograph of (a) H and E, ×40 section and (b) immunohistochemistry, ×100 of CD 20 from tumor showing monotonous population of small round cells with scanty cytoplasm, coarse nuclear chromatin and prominent nucleoli in the pancreatic parenchyma

Hematoxylin and eosin-stained sections from tumor in all the 3 patients showed monotonous population of small round cells in sheets separated by sparse fibrous septae infiltrating the pancreatic parenchyma [Figure 2]. Neoplastic cells had scanty cytoplasm, coarse nuclear chromatin and prominent nucleoli. On immunohistochemistry, all the tumors were positive for CD 20 and negative for CD 3 and were typed as a diffuse large B cell lymphoma of the pancreas.

Two patients received chemotherapy (cyclophosphamide; adriamycin hydrochloride; vincristine and predinosolone — CHOP regimen) for 4 cycles followed by radiotherapy (45 Gy in 20 fractions for 1 patient and 30 Gy in 18 fractions for the other) using antero-posterior and postero-anterior pair beams. One patient who was diagnosed following Whipple′s procedure received adjuvant chemotherapy with CHOP regimen (6 cycles) alone.

All the patients are currently on follow-up (range, 1-36 months) and are so far free of disease recurrence.

## DISCUSSION

Diagnostic criteria for PPL include presence of main mass in the pancreas with lymph nodal involvement confined to the peri-pancreatic region with no hepatic or splenic involvement; absence of superficial lymphadenopathy and mediastinal lymph node enlargement; and a normal leukocyte count in peripheral blood.[8,9]

Literature suggests a strong male predominance (male-to-female ratio, 7:1).[6,10]There is no significant difference noted with regard to patient′s age or duration of symptoms between pancreatic adenocarcinoma and PPL.[11-13]

Most common presenting symptom reported in literature is abdominal pain, found in about 83% of cases, followed by (in order of frequency) abdominal mass (58%), weight loss (50%), jaundice (37%), acute pancreatitis (12%), small-bowel obstruction (12%) and diarrhea (12%).[1,3,6,8,9] Obstructive jaundice seems to be less frequent in PPL than in pancreatic adenocarcinoma.[8,9] In this series, the most common presenting symptom was vague abdominal pain, as reported previously. One of our patients had low-grade fever, while another had melena, both of which have not been described in this setting.

Usual location of the tumor mass is in the head of the pancreas (more than 80%), with size ranging from 2 to 15 cm.[10,14] In our series, body of the pancreas was the site of disease for all the 3 patients, which is a rare occurrence, with 1 patient having involvement of head and tail of the pancreas.

PPL should be suspected if imaging shows a bulky localized tumor in the pancreatic head without significant dilatation of the main pancreatic duct, infrahilar retroperitoneal enlarged lymph nodes and invasive tumor growth not respecting anatomic boundaries with infiltration of surrounding structures. Enhancement after administration of intravenous contrast medium is usually poor yet homogeneous.[15] Presence of calcification or necrosis is usually indicative of pathology other than non-Hodgkin′s lymphoma.

An accurate guided core biopsy for confirmation of diagnosis of PPL is considered a safe, rapid and easy procedure with high diagnostic accuracy.[16,17] We were able to avoid unnecessary laparotomy in 2 patients.

All cases of PPL reported to date in western countries are of the B-cell type, but some cases of T-cell pancreatic lymphoma have been described in Japanese series.[1,3,5,8,10,12,13] In our series, all the 3 cases were diffuse large B-cell type, akin to those described in western literature.

Tumor burden, beta-2-microglobulin level above 2 mg/L and LDH level higher than normal are poor prognostic markers.[1,9,14,19] Anecdotal cases of elevated CA 19-9 levels in PPL have been reported.[18]

Chemotherapy, similar to that for non-Hodgkin′s lymphoma, is the present standard treatment for most patients with PPL, with cure rates of up to 30%.[1,3–6,10,16,19]

The role of surgery is limited to rare occasions such as when FNA or core biopsy is nondiagnostic and open biopsy, therefore, is required.[20] Total pancreatectomy (Whipple′s procedure) is considered to have no impact on survival in a patient with PPL and, with its associated morbidity, is not generally recommended for PPL.[16]

The role of radiation therapy in the management of PPL is not yet defined. Local radiotherapy up to total 40 Gy has been used as consolidation,[21] but data is sparse to draw any conclusions.

Larger series of patients are needed to evaluate whether chemotherapy, eventually followed by involved-field radiation therapy, is the treatment of choice for PPL [Table 1].[10,13]

**Table 1:** Clinical and laboratory data of the 3 patients of primary pancreatic lymphoma

Parameter	Case I	Case II	Case III	Reference range
Age	65	63	52	
Sex	Female	Female	Male	
Duration of symptoms	3 months	2 months	1.5 months	
LDH	408.1	240	173.2	0-240 IU/L
Bilirubin (total)	2	4.2	6	0.2-1.0 mg/dL
Alkaline phosphatase	98	121	128	39-117 IU/L
SGOT	42	58	71	5-38 IU/L
Amylase	68	78	143.5	28-100 IU/L
CA 19-9	78.1	111.8	62.2	0-37 IU/L
Diagnostic modality	CT scan and guided biopsy	CT scan and guided biopsy	Surgery (Whipple’s procedure)	
Chemotherapy	CHOP regimen	CHOP regimen	Adjuvant CHOP	
Radiotherapy	45 Gy in 20 fractions	30 Gy in 18 fractions	Planned	
Follow-up duration*	36 months	25 months	1 month	
Status at last follow-up	NED	NED	NED	

CHOP regimen = Cyclophosphamide, doxorubicin hydrochloride, vincristine and predinosolone; NED = No evidence of disease;

* = Since completion of chemotherapy

## References

[1] Zucca E, Roggero E, Bertoni F, Cavalli F (1997). Primary extranodal non-Hodgkin′s lymphomas: Part 1: Gastrointestinal, cutaneous and genitourinary lymphomas. Ann Oncol.

[2] Joly I, David A, Payan MJ, Sahel J, Sarles H (1992). A case of primary non-Hodgkin′s lymphoma of the pancreas. Pancreas.

[3] Boni L, Benevento A, Dionigi G, Cabrini L, Dionigi R (2002). Primary pancreatic lymphoma. Surg Endosc.

[4] Ezzat A, Jamshed A, Khafaga Y, Rahal M, Linjawi T, Martin J (1996). Primary pancreatic non-Hodgkin′s lymphomas. J Clin Gastroenterol.

[5] Baylor SM, Berg JW (1973). Cross-classification and survival characteristics of 5,000 cases of cancer of the pancreas. J Surg Oncol.

[6] Saif MW (2006). Primary pancreatic Lymphomas. J Pancreas.

[7] Chakravarty U, Vartak S, Chodankar C, Ranadive N (2005). Primary pancreatic lymphoma. Bombay Hosp J.

[8] Dawson IM, Cornes JS, Morson BC (1961). Primary malignant lymphoid tumours of the intestinal tract: Report of 37 cases with a study of factors influencing prognosis. Br J Surg.

[9] Behrns KE, Sarr MG, Strickler JG (1994). Pancreatic lymphoma: Is it a surgical disease?. Pancreas.

[10] Nayer H, Weir EG, Sheth S, Ali SZ (2004). Primary pancreatic lymphoma. Cancer.

[11] James JA, Milligan DW, Morgan GJ, Crocker J (1998). Familial pancreatic lymphoma. J Clin Pathol.

[12] Nishimura R, Takakuwa T, Hoshida Y, Tsujimoto M, Aozasa K (2001). Primary pancreatic lymphoma: Clinicopathological analysis of 19 cases from Japan and review of the literature. Oncology.

[13] Arcari A, Anselmi E, Bernuzzi P, Berte R, Lazzaro A, Moroni CF (2005). Primary pancreatic lymphoma: Report of five cases. Haematologica.

[14] Islam S, Callery MP (2001). Primary pancreatic lymphoma: A diagnosis to remember. Surgery.

[15] Merkle EM, Bender GN, Brambs HJ (2000). Imaging findings in pancreatic lymphoma: Differential aspects. Am J Roentgenol.

[16] Tuchek JM, De Jong SA, Pickleman J (1993). Diagnosis, surgical intervention, and prognosis of primary pancreatic lymphoma. Am Surg.

[17] Faulkner JE, Gaba CE, Powers JD, Yam LT (1998). Diagnosis of primary pancreatic lymphoma by fine needle aspiration. Acta Cytol.

[18] Kawakami K, Nomura H, Watanabe Y, Momma F (2002). Primary pancreatic lymphoma with elevated serum CA19-9 level. Rinsho Ketsueki.

[19] Tondini C, Giardini R, Bozzetti F, Valagussa P, Santoro A, Bertulli R (1993). Combined modality treatment for primary gastrointestinal non-Hodgkin′s lymphoma: The Milan Cancer Institute experience. Ann Oncol.

[20] Mansour GM, Cucchiaro G, Niotis MT, Fetter BF, Moore J, Rice RR (1989). Surgical management of pancreatic lymphoma. Arch Surg.

[21] Shahar KH, Carpenter LS, Jorgensen J, Truong L, Baker K, Teh BS (2005). Role of radiation therapy in a patient with primary pancreatic lymphoma. Clin Lymph Myeloma.

